# Ultrapotent IgA dimeric antibodies neutralize emerging Omicron variants

**DOI:** 10.1128/jvi.01740-24

**Published:** 2024-12-04

**Authors:** Fanglei Zuo, Yunlong Cao, Rui Sun, Qianran Wang, Luca Simonelli, Likun Du, Federico Bertoglio, Maren Schubert, Concetta Guerra, Andrea Cavalli, Michael Hust, Davide F. Robbiani, Luca Varani, Hassan Abolhassani, Xiaoliang Sunney Xie, Lennart Hammarström, Harold Marcotte, Qiang Pan-Hammarström

**Affiliations:** 1 Division of Immunology, Department of Medical Biochemistry and Biophysics, Karolinska Institutet 344286 , Stockholm, Sweden; 2 Biomedical Pioneering Innovation Center (BIOPIC), Peking University 593075 , Beijing, China; 3 Changping Laboratory 662243 , Beijing, China; 4 Institute for Research in Biomedicine, Università della Svizzera italiana 27216 , Bellinzona, Switzerland; 5 Institute of Biochemistry, Biotechnology and Bioinformatics, Department of Medical Biotechnology, Technische Universität Braunschweig 26527 , Braunschweig, Germany; St. Jude Children's Research Hospital, Memphis, Tennessee, USA

**Keywords:** SARS-CoV-2, neutralizing antibodies, dimeric IgA, immune escape

## LETTER

The severe acute respiratory syndrome coronavirus 2 (SARS-CoV-2) JN.1 strain, which became globally predominant in the first half of 2024, has an additional L455S mutation in its receptor-binding domain compared to its precursor, BA.2.86, enhancing its ability to evade immune responses (1–3). Some subvariants derived from JN.1, such as KP.2 and KP.3, exhibit even greater immune escape abilities (4, 5). The reduced antibody response to JN.1 and its derivative variants is likely responsible for the increase in infections and hospitalizations in summer 2024 (6). Given concerns about immunological imprinting and the lack of an effective mucosal vaccine (7), administering cross-reactive monoclonal antibodies (mAbs) at the mucosal surface as pre-exposure prophylaxis may offer alternative protection.

**Fig 1:**
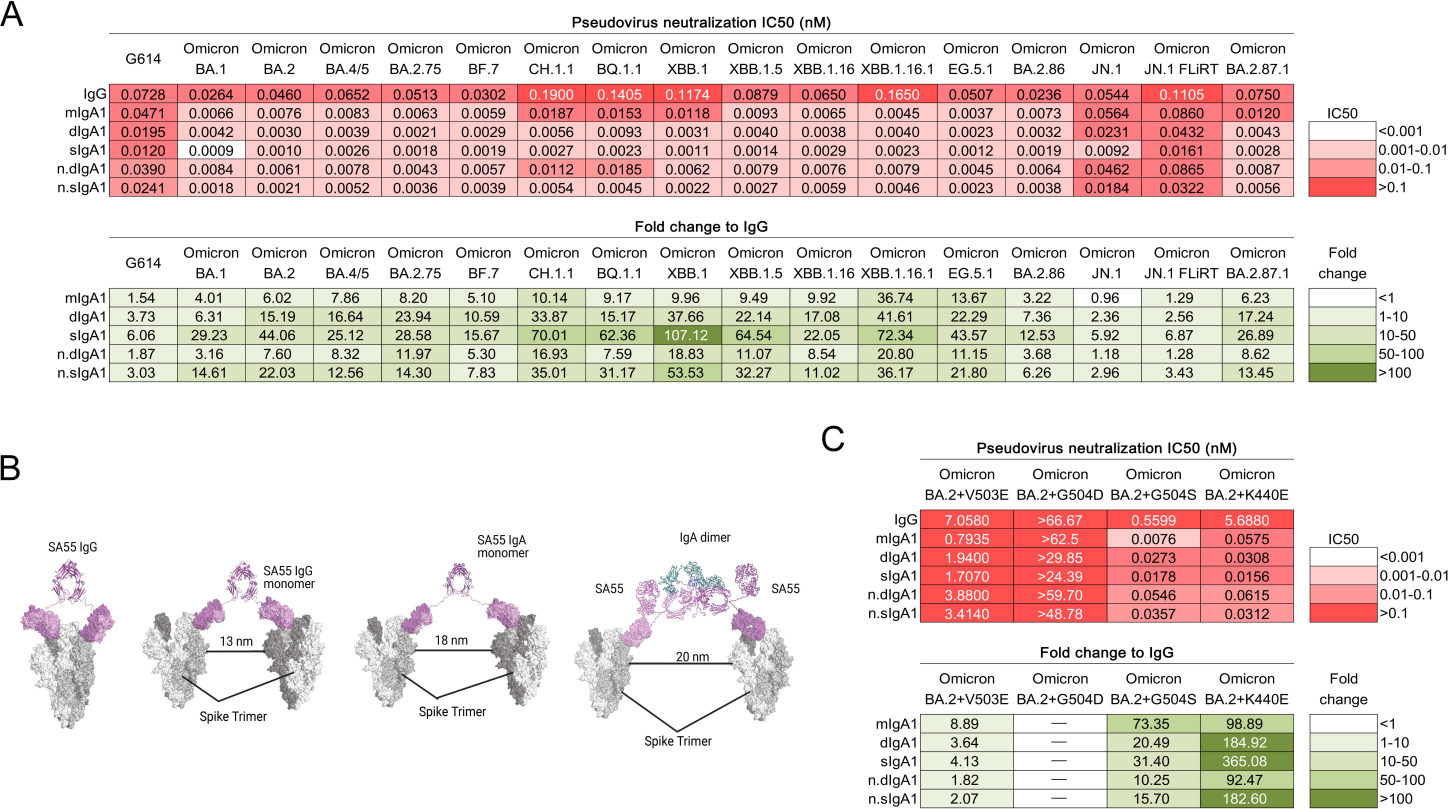
(**A**) SA55 monomeric IgA1, dimeric IgA1, and secretory IgA1 showed enhanced neutralization activity against pseudotyped Omicron variants compared to their IgG counterpart. (**B**) Computational simulation showing intra-spike linking by SA55 monomeric IgG and inter-spike linking by SA55 monomeric IgG, monomeric IgA1, and dimeric IgA1 antibodies. The predicted maximum distance between S-trimers, which allows for interlinking, is indicated. (**C**) SA55 mIgA1, dIgA1, and sIgA1 enhanced or restored the neutralization activity against pseudotyped mutants BA.2+K440E, BA.2+V503E, and BA.2+G504S. The half-maximal inhibitory concentration (IC50) and fold-change differences between IgG and IgA1 antibody forms are indicated. n.dIgA1 and n.sIgA1, normalized values according to the number of binding sites.

We previously showed that converting recombinant monoclonal IgG into dimeric and secretory IgA can significantly enhance neutralization against Omicron variants, with dimeric IgA providing protection in a humanized mouse model when administered intranasally (8). Here, we engineered the potent cross-reactive neutralizing antibody, SA55, originally isolated from a SARS-CoV-2-vaccinated and SARS-CoV-1 convalescent individual (9), into monomeric IgA1 (mIgA1), dimeric IgA1 (dIgA1), and secretory IgA1 (sIgA1) formats, and compared their neutralization activity against pseudotyped viruses. SA55 IgG neutralized all tested Omicron variants, including JN.1, JN.1 FLiRT, and the highly mutated BA.2.87.1 (10), with half-maximal inhibitory concentration (IC50) values ranging from 0.02 to 0.19 nM, respectively. Compared to IgG, mIgA1 increased neutralizing activities against Omicron variants by up to 36.7-fold, while dIgA1 and sIgA1 increased the neutralizing activity by up to 53.5-fold (Fig. 1A; Fig. S1). The docking model shows that SA55 IgG can engage in both intra- and inter-spike linking, but conversion to mIgA1 and dIgA1 significantly enhances inter-spike binding, likely due to greater hinge flexibility and wider arm distance (Fig. 1B) (8).

Since the increase in neutralization activity against a particular SARS-CoV-2 variant by conversion to IgA1 was more pronounced when the parental IgG exhibited lower (but detectable) neutralization activity (higher IC50) (8), we evaluated the neutralization efficacy of SA55 IgA1 antibodies against the pseudotyped mutants BA.2+K440E, BA.2+V503E, BA.2+G504D, and BA.2+G504S, which exhibit notable resistance to SA55 IgG (9). Indeed, conversion to IgA1 strongly enhanced or restored the neutralization potency of SA55 against these mutants (8.9- to 99-fold for mIgA1 and 1.8- to 183-fold for dIgA1 and sIgA1), except BA.2+G504D, which was not neutralized by SA55 IgG (Fig. 1C). Although mutations at positions V503 and G504 are rarely observed in circulating SARS-CoV-2 variants (9), SA55 IgA1 may remain effective against potential emerging variants harboring these mutations.

Since the emergence of the BQ and XBB lineages in late 2022, approved therapeutic mAbs have either become less effective or lost their neutralizing activity (11, 12). In contrast, SA55 in IgA1 formats can neutralize all known prevalent variants, including JN.1. With an IC50 ranging from 0.002 to 0.087 nM, its potency is comparable to or exceeds that of clinically approved antibodies used against current and previous variants (Table S1). This antibody has the potential to be developed as an innovative immunotherapeutic for short-term prophylaxis through intranasal delivery (8).

## SUPPLEMENTAL MATERIAL

Supplemental material jvi.01740-24-s0001.docx Supplemental methods, Fig. S1, and Table S1.

ASM does not own the copyrights to Supplemental Material that may be linked to, or accessed through, an article. The authors have granted ASM a non-exclusive, world-wide license to publish the Supplemental Material files. Please contact the corresponding author directly for reuse.
