# Supplementary material for: Ultrapotent IgA dimeric antibodies neutralize emerging Omicron variants
Source: J Virol. 2024 Dec 4;99(1):e01740-24. doi: 10.1128/jvi.01740-24 (PMC11784158; doi:10.1128/jvi.01740-24)
Supplement: Supplemental material — Supplemental methods, Fig. S1, and Table S1. [file jvi.01740-24-s0001.docx]

Supplementary Materials for

Ultrapotent IgA dimeric antibodies neutralize emerging Omicron variants

Fanglei Zuo^1^, Ph.D., Yunlong Cao^2,3^, Ph.D., Rui Sun^1^, M.S., Qianran Wang^2,3^, Ph.D., Luca Simonelli^4^, Ph.D., Likun Du^1^ Ph.D., Federico Bertoglio^5^, Ph.D., Maren Schubert^5^, Ph.D., Concetta Guerra^4^, Ph.D., Andrea Cavalli^4^, Ph.D., Michael Hust^5^, Ph.D., Davide F. Robbiani^4,^ M.D., Ph.D., Luca Varani^4^, Ph.D., Hassan Abolhassani^1^, M.D., Ph.D., Xiaoliang Sunney Xie^2,3^, Ph.D., Lennart Hammarström^1^, M.D., Ph.D., Harold Marcotte^1#^, Ph.D., Qiang Pan-Hammarström^1#^, M.D., Ph.D.

Correspondence to: qiang.pan-hammarstrom@ki.se

# **Materials and Methods**

## ***Production and purification of antibodies***

The IgG and IgA1 (including monomeric [mIgA1], dimeric [dIgA1], and secretory [sIgA1] formats) SA55 antibodies were produced as previously described (GenScript) (1). The IgG and IgA1 antibodies were purified by single-step affinity chromatography using immobilised protein A (AmMagTM Ultra AT Protein A MagBeads) or anti-IgA antibody (CaptureSelect™ IgA Affinity Matrix), respectively (GenScript).

## ***Pseudovirus neutralization assay***

The human codon-optimized gene encoding the S protein of G614, BA.1, BA.2, BA.4/5, BF.7, CH.1.1, BA.2.86, and BA.2.87.1, lacking the C-terminal 19 codons (S_Δ19_), was synthesized by GenScript (1-4). Site-directed mutagenesis (QuikChange Multi Site-Directed Mutagenesis Kit, Agilent) was utilized to construct the S_Δ19_ gene of BA.2.75, BQ.1.1, XBB/XBB.1, XBB.1.5, XBB.1.16, XBB.1.16.1, EG.5.1, JN.1, JN.1 FLiRT (JN.1+R346T+F456L), BA.2+K440E, BA.2+V503E, BA.2+G504D, and BA.2+G504S variants, using the BA.2, BA.4/5, or BA.2.86 S_Δ19_ gene as a template (1-3, 5). S pseudotyped virus was produced based on a pseudotyped HIV virus production system, as previously described (1, 6).

The pseudovirus neutralization assay was performed as previously described (1). Each antibody dilution was tested in duplicate, and the mean neutralization (%) values are presented in the figures. The IC50 values for the monoclonal antibodies were determined using four-parameter nonlinear regression (GraphPad Prism 7.04 software) (1).

## ***Computational modelling***

Computational structure modelling of SA55 was performed based on crystallization of the antibody with Omicron BA.1 RBD complex (Protein Data Bank (PDB) ID: 7Y0W). The structures of monomeric IgG, mIgA1 and dIgA1 SA55 bound to two SARS-CoV-2 S trimers were built using PyMOL software (The PyMOL Molecular Graphics System, Version 2.0 Schrödinger, LLC).

# **References**

1. Marcotte H, Cao Y, Zuo F, et al. Conversion of monoclonal IgG to dimeric and secretory IgA restores neutralizing ability and prevents infection of Omicron lineages. Proc Natl Acad Sci. 2024;121(3):e2315354120. doi.org/10.1073/pnas.2315354120.

2. Bianchini F, Crivelli V, Abernathy M, et al. Human neutralizing antibodies to cold linear epitopes and subdomain 1 of the SARS-CoV-2 spike glycoprotein. Sci Immunol. 2023;8:eade0958. doi.org/10.1126/sciimmunol.ade0958.

3. Cao Y, Jian F, Wang J, et al. Imprinted SARS-CoV-2 humoral immunity induces convergent Omicron RBD evolution. Nature. 2023;614:521-529. doi.org/10.1038/s41586-022-05644-7.

4. Yang S, Yu Y, Jian F, et al. Antigenicity assessment of SARS-CoV-2 saltation variant BA.2.87.1. Emerg Microbes Infect. 2024;13:2343909. doi.org/10.1080/22221751.2024.2343909.

5. Cao Y, Jian F, Zhang Z, et al. Rational identification of potent and broad sarbecovirus-neutralizing antibody cocktails from SARS convalescents. Cell Rep. 2022;41:111845. doi.org/10.1016/j.celrep.2022.111845.

6. Schmidt F, Weisblum Y, Muecksch F, et al. Measuring SARS-CoV-2 neutralizing antibody activity using pseudotyped and chimeric viruses. J Exp Med. 2020; 217(11):e20201181. doi.org/10.1084/jem.20201181.

**
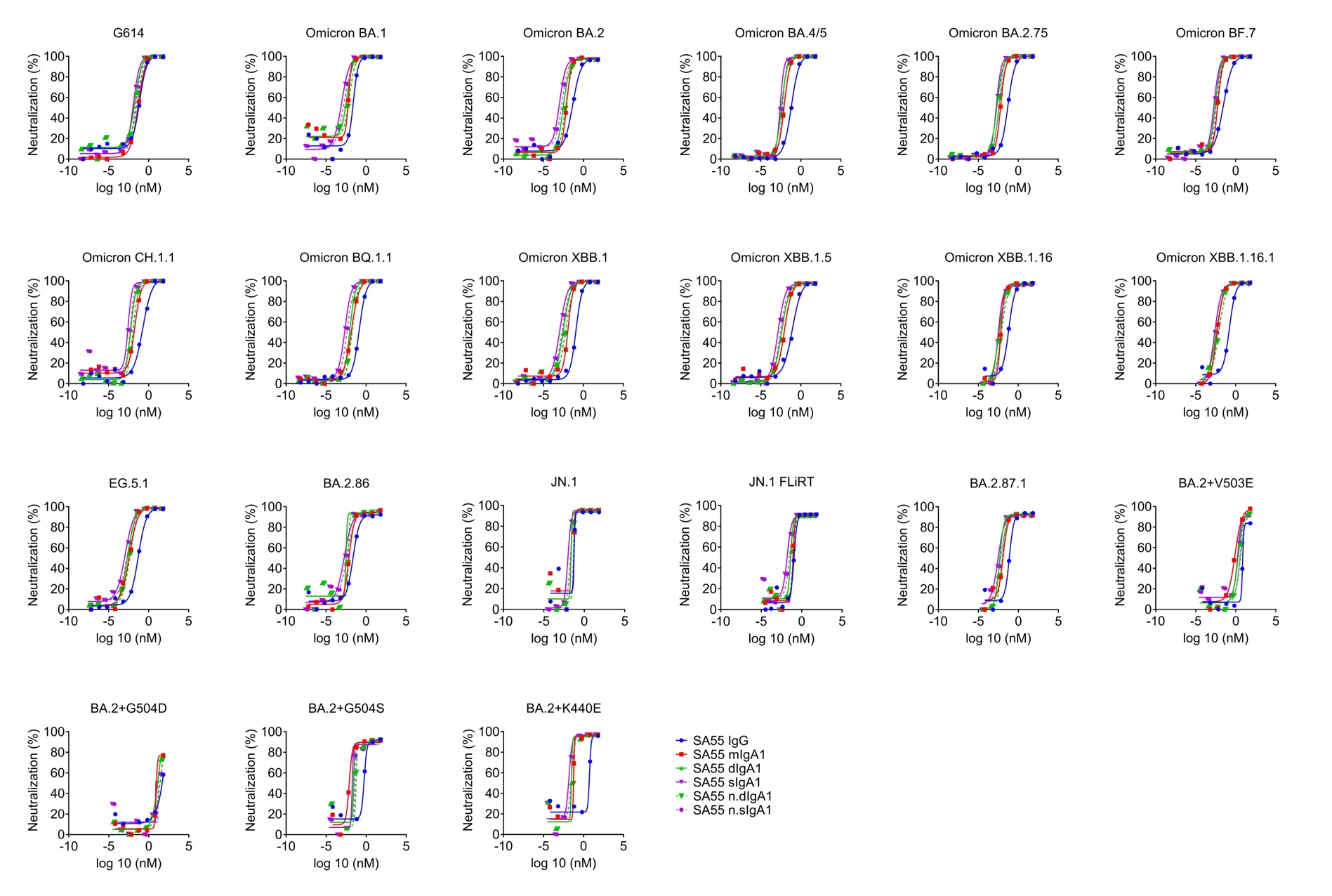
**

**Supplementary Fig. 1.** SA55 monomeric IgA1 (mIgA1), dimeric IgA1 (dIgA1), and secretory IgA1 (sIgA1) showed enhanced neutralization activity against pseudotyped Omicron variants compared to its IgG counterpart. Each antibody dilution was run in duplicate, and the mean neutralization (%) values are presented. n.dIgA1 and n.sIgA1 represent normalized values according to the number of binding sites.

**Supplementary Table 1.** Comparison of SA55-IgG and IgA1 neutralization activity with commercially available antibodies and antibodies described in literature.

| **Antibodies** | **Pseudovirus neutralisation activity (IC50 (pM) normalized based on the number of binding sites)** | | | | | | | | | | | | | | | | | **Refer-ences**^a^ |  |
| --- | --- | --- | --- | --- | --- | --- | --- | --- | --- | --- | --- | --- | --- | --- | --- | --- | --- | --- | --- |
|  | G614 | BA.1 | BA.2 | BA.4/5 | BA.2.75 | BF.7 | CH.1.1 | BQ.1.1 | XBB.1 | XBB.1.5 | XBB.1.16 | XBB.1.16.1 | EG.5.1 | BA.2.86 | JN.1 | JN.1 FLiRT^b^ | BA.2.87.1 |  |  |
| SA55 IgG | 72,8 | 26,4 | 46 | 65,2 | 51,3 | 30,2 | 190 | 140,5 | 117,4 | 87,9 | 65 | 165 | 50,7 | 23,6 | 54,4 | 110,5 | 75 | This paper |  |
| SA55 mIgA1 | 47,1 | 6,6 | 7,6 | 8,3 | 6,3 | 5,9 | 18,7 | 15,3 | 11,8 | 9,3 | 6,5 | 4,5 | 3,7 | 7,3 | 56,4 | 86 | 12 | This paper |  |
| SA55 dIgA1 | 39 | 8,4 | 6,1 | 7,8 | 4,3 | 5,7 | 11,2 | 18,5 | 6,2 | 7,9 | 7,6 | 7,9 | 4,5 | 6,4 | 46,2 | 86,5 | 8,7 | This paper |  |
| SA55 sIgA1 | 24,1 | 1,8 | 2,1 | 5,2 | 3,6 | 3,9 | 5,4 | 4,5 | 2,2 | 2,7 | 5,9 | 4,6 | 2,3 | 3,8 | 18,4 | 32,2 | 5,6 | This paper |  |
| DXP-604 IgG | 112,9 | 120,5 | 90,9 | 835,5 | 1016 | ND | ND | 8753 | >66670 | ND | ND | ND | ND | ND | ND | ND | ND | 1 |  |
| DXP-604 mIgA1 | 100,7 | 8,9 | 6,9 | 22,1 | 30,4 | ND | ND | 5767 | >62500 | ND | ND | ND | ND | ND | ND | ND | ND | 1 |  |
| DXP-604 dIgA1 | 74,8 | 3,4 | 1,8 | 22 | 55 | ND | ND | 9754 | >59700 | ND | ND | ND | ND | ND | ND | ND | ND | 1 |  |
| DXP-604 sIgA1 | 65,4 | 1,6 | 1,2 | 7,6 | 26,1 | ND | ND | 3182 | >48780 | ND | ND | ND | ND | ND | ND | ND | ND | 1 |  |
| 01A05 IgG | 28 | ND | ND | ND | 419,5 | ND | ND | >66670 | >66670 | 30480 | 7518 | ND | ND | ND | ND | ND | ND | 1 |  |
| 01A05 mIgA1 | 14 | ND | ND | ND | 12,7 | ND | ND | >62500 | 300 | 115,4 | 79,1 | ND | ND | ND | ND | ND | ND | 1 |  |
| 01A05 dIgA1 | 9,6 | ND | ND | ND | 4,9 | ND | ND | >59700 | 269,4 | 131,6 | 62,8 | ND | ND | ND | ND | ND | ND | 1 |  |
| 01A05 sIgA1 | 9,1 | ND | ND | ND | 2,3 | ND | ND | >48780 | 350,8 | 105,6 | 76,2 | ND | ND | ND | ND | ND | ND | 1 |  |
| LY-CoV1404 (bebtelovimab,  Eli Lilly) | 4 | 4 | 6 | 6 | 2,2 | ND | >33350 | >33350 | >33350 | >66670 | ND | ND | >66670 | >66670 | >66670 | ND | >66670 | 2-7 |  |
| S309/ Sotrovimab | 493,3 | 2410 | 6120 | 5280 | 980 | 1520 | 711 | >33350 | 965 | 575 | 245 | ND | 192 | 12606 | 15341 | 15800 | 3735 | 2,4,5, 8-10 |  |
| COV2-2130 (Cilgavimab) | 16,7 | 20050 | 42 | 153 | 480 | ND | >33350 | >33350 | >33350 | >66670 | ND | ND | >66670 | >66670 | ND | ND | >66670 | 2,4,6,10 |  |
| REGN10987 (Regeneron) | 38 | >66670 | 3930 | 3470 | >33350 | ND | >33350 | ND | >33350 | ND | ND | ND | ND | ND | ND | ND | >66670 | 2,11 |  |
| REGN10933+ REGN10987 (Regeneron) | 33,3 | >66670 | 5470 | 4730 | 63325 | ND | >33350 | ND | >33350 | ND | ND | ND | ND | ND | ND | ND | ND | 2,11 |  |
| AZD1061  (Astra Zeneca) | 13,3 | 2050 | 53,3 | 100 | 2348 | ND | >66670 | >66670 | >66670 | >66670 | ND | ND | >33350 | >33350 | ND | ND | ND | 12-16 |  |
| AZD7442  (Astra Zeneca) (Tixagevimab+  Cilgavimab) | 6,7 | 1550 | 53,3 | 433 | 807,1 | ND | >66670 | >66670 | >66670 | >66670 | >66670 | ND | >66670 | >33350 | ND | ND | ND | 12,14-17 |  |
|  |  |  |  |  |  |  |  |  |  |  |  |  |  |  |  |  |  |  |  |
| AZD3152 (Sipavibart, Astra Zeneca) | 90 | 36 | 71,4 | 31,3 | 166,8 | 25,3 | ND | 61,4 | 24 | 21,3 | 36 | ND | 6151 | 44 | 642,3 | ND | ND | 18 |  |
| VYD222 (Pemivibart, Permagard) | 56 | 810 | 298 | 319,5 | 9102 | 484,9 | ND | 618 | 807,7 | 695,7 | 517,6 | ND | ND | 1119 | 497,6 | ND | ND | 19 |  |
| ^a^ References are listed below. | | | | | | | | | | | | | | | | | | |  |
| ^b^ JN.1 FLiRT, JN.1 that carries the R346T and F456L spike mutations. | | | | | | | | | | | | | | | | | | |  |
| ^c^ ND, not determined. | | | | | | | | | | | | | | | | | | |  |
|  |  |  |  |  |  |  |  |  |  |  |  |  |  |  |  |  |  |  |  |
| **References** | |  |  |  |  |  |  |  |  |  |  |  |  |  |  |  |  |  |  |
| 1. Marcotte H, Cao Y, Zuo F, et al. Conversion of monoclonal IgG to dimeric and secretory IgA restores neutralizing ability and prevents infection of Omicron lineages. Proc Natl Acad Sci. 2024;121(3):e2315354120. doi.org/10.1073/pnas.2315354120. | | | | | | | | | | | | | | | | | | |  |
| 2. Cao Y, Yisimayi A, Jian F, et al. BA.2.12.1, BA.4 and BA.5 escape antibodies elicited by Omicron infection. Nature. 2022;608:593-602. doi.org/10.1038/s41586-022-04980-y. | | | | | | | | | | | | | | | | | | |  |
| 3. Wang Q, Guo Y, Zhang R, et al. Antibody neutralisation of emerging SARS-CoV-2 subvariants: EG.5.1 and XBC.1.6. Lancet Infect Dis. 2023;23:E397-E398. doi.org/10.1016/S1473-3099(23)00555-8. | | | | | | | | | | | | | | | | | | |  |
| 4. Wang Q, Guo Y, Liu L, et al. Antigenicity and receptor affinity of SARS-CoV-2 BA.2.86 spike. Nature. 2023;624:639-644. doi.org/10.1038/s41586-023-06750-w. | | | | | | | | | | | | | | | | | | |  |
| 5. Arora P, Kempf A, Nehlmeier I, et al. Omicron sublineage BQ.1.1 resistance to monoclonal antibodies. Lancet Infect Dis. 2023;23(1):22-23. doi.org/10.1016/S1473-3099(22)00733-2. | | | | | | | | | | | | | | | | | | |  |
| 6. Ju B, Guo H, Wang M, et al. Striking antibody evasion of SARS-CoV-2 Omicron sub-lineages BQ. 1.1, XBB.1 and CH.1.1. Nati Sci Rev. 2023;10(8):nwad148. doi.org/10.1093/nsr/nwad148. | | | | | | | | | | | | | | | | | | |  |
| 7. Yang S, Yu Y, Jian F, et al. Antigenicity assessment of SARS-CoV-2 saltation variant BA.2.87.1. Emerg Microbes Infect. 2024;13:2343909. doi.org/10.1080/22221751.2024.2343909. | | | | | | | | | | | | | | | | | | |  |
| 8. Liu L, Casner R, Guo Y, et al. Antibodies targeting a quaternary site on SARS-CoV-2 spike glycoprotein prevent viral receptor engagement by conformational locking. Immunity. 2023;56(10):2442-2455. doi:10.1016/j.immuni.2023.09.003. | | | | | | | | | | | | | | | | | | |  |
| 9. Wang Q, Mellis I, ho J, et al. Recurrent SARS-CoV-2 spike mutations confer growth advantages to select JN.1 sublineages. Emerg Microbes Infect. 2024;3:2402880. doi.org/10.1080/22221751.2024.2402880. | | | | | | | | | | | | | | | | | | |  |
| 10. Yang S, Yu Y, Xu Y, et al. Fast evolution of SARS-CoV-2 BA.2.86 to JN.1 under heavy immune pressure. Lancet Infect Dis. 2024;24(2):E70-E72. doi.org/10.1016/S1473-3099(23)00744-2. | | | | | | | | | | | | | | | | | | |  |
| 11. Uraki R, Ito M, Kiso M, et al. Efficacy of antivirals and bivalent mRNA vaccines against SARS-CoV-2 isolate CH. 1.1. Lancet Infect Dis. 2023;23(5):525-526. doi.org/10.1016/S1473-3099(23)00132-9. | | | | | | | | | | | | | | | | | | |  |
| 12. Case J, Mackin S, Errico J, et al. Resilience of S309 and AZD7442 monoclonal antibody treatments against infection by SARS-CoV-2 Omicron lineage strains. Nat Commun. 2022;13:3824. doi.org/10.1038/s41467-022-31615-7. | | | | | | | | | | | | | | | | | | |  |
| 13. Tuekprakhon A, Nutalai R, Dijokaite-Guraliuc A, et al. Antibody escape of SARS-CoV-2 Omicron BA.4 and BA.5 from vaccine and BA.1 serum. Cell. 2022;185:2422-2433. doi.org/10.1016/j.cell.2022.06.005. | | | | | | | | | | | | | | | | | | |  |
| 14. Xue S, Huan Y, Wu F, et al. Mutations in the SARS-CoV-2 spike receptor binding domain and their delicate balance between ACE2 affinity and antibody evasion. Protein *&* Cell. 2024;15(6):403-418. doi.org/10.1093/procel/pwae007. | | | | | | | | | | | | | | | | | | |  |
| 15. Zhang L, Kempf A, Nehlmeier I, et al. SARS-CoV-2 BA.2.86 enters lung cells and evades neutralizing antibodies with high efficiency. Cell. 2024;187(3):596-608. doi.org/10.1016/j.cell.2023.12.025. | | | | | | | | | | | | | | | | | | |  |
| 16. Lee J, Naoe, Y, Bang U, et al. Neutralization sensitivity of SARS-CoV-2 Omicron variants FL.1 and GE.1 by therapeutic antibodies and XBB sera. Virology. 2024;595:110067. doi.org/10.1016/j.virol.2024.110067. | | | | | | | | | | | | | | | | | | |  |
| 17. Zhang L, Kempf A, Nehlmeier I, et al. Neutralisation sensitivity of SARS-CoV-2 lineages EG.5.1 and XBB.2.3. Lancet Infect Dis. 2023;23(10):E391-E392. doi.org/10.1016/S1473-3099(23)00547-9.  18. Cai Y, Diallo S, Rosenthal K, et al. AZD3152 neutralizes SARS-CoV-2 historical and contemporary variants and is protective in hamsters and well tolerated in adults. Sci Transl Med. 2024;16(753):eado2817. doi.org/10.1126/scitranslmed.ado2817.  19. CDER Scientific Review Documents Supporting Emergency Use Authorizations for Drug and Biological Therapeutic Products \| COVID-19. 2024. (Accessed 08/22/2024, at https://www.fda.gov/drugs/coronavirus-covid-19-drugs/cder-scientific-review-documents-supporting-emergency-use-authorizations-drug-and-biological.) | | | | | | | | | | | | | | | | | | |  |
